# Frailty in Old Age Is Associated with Decreased Interleukin-12/23 Production in Response to Toll-Like Receptor Ligation

**DOI:** 10.1371/journal.pone.0065325

**Published:** 2013-06-05

**Authors:** Nathalie Compté, Karim Zouaoui Boudjeltia, Michel Vanhaeverbeek, Sandra De Breucker, Joel Tassignon, Anne Trelcat, Thierry Pepersack, Stanislas Goriely

**Affiliations:** 1 Institute for Medical Immunology (IMI), Université Libre de Bruxelles, Charleroi, Belgium; 2 Experimental Medicine Laboratory (Unit 222), Université Libre de Bruxelles, Hôpital A. Vésale, Montigny-Le-Tilleul, Belgium; 3 Service de gériatrie, Hôpital Erasme, Bruxelles, Belgium; 4 ImmuneHealth, Charleroi, Belgium; University of Medicine and Dentistry of New Jersey - New Jersey Medical School, United States of America

## Abstract

Aging is associated with progressive alterations of immune functions, leading to higher susceptibility to bacterial and viral infections and reduced vaccine responses. Data concerning cytokine production in response to Toll-like receptor (TLR) ligands are highly variable in old people, reflecting the heterogeneity of the geriatric population. The aim of our study was to define the relative contribution of age and clinical status on TLR-induced interleukin (IL)-12p70 and IL-23 production as these cytokines play an important role in the protection against intracellular and extracellular pathogens, respectively. For this purpose, we recruited 100 subjects (aged 23–96 years) in the general population or hospitalized for chronic diseases. We collected information on clinical status (medical history, ongoing comorbidities, treatments and geriatric scales), biological parameters (biochemical and hematological tests, telomere length determination, cytomegalovirus serology). Whole blood samples were stimulated with a combination of TLR4 and TLR7/8 ligands. We performed univariate and stepwise backward multivariate analyses regression to define which set of clinical variables could be predictive for IL-12p70 and IL-23 production in these conditions. Our results indicated that age was not correlated with TLR-mediated IL-12p70 and IL-23 production. In contrast, poor nutritional status and frailty in subjects >75 years were associated with decreased IL-12p70 and IL-23 production. By intracytoplasmic staining, we confirmed that production of IL-12/23p40 by conventional dendritic cells (DCs) upon TLR ligation was decreased in frail patients. However, proportion of DCs and monocytes subsets, phenotypic maturation and proximal signaling events were found to be comparable in frail and healthy old subjects. These results suggest the importance of age-associated clinical parameters and not age by itself in the alteration of innate immune responses in old individuals and emphasis the importance of innate immune responses in the susceptibility of frail geriatric patients to infections.

## Introduction

Demographic evolution represents a challenge for public health. Global population, especially in the developed countries is aging. The proportion of the population above 60 years has increased from 8% in 1950 to 10% in 2000 and is expected to reach 21% by 2050 [1]. Decline in immune function is a hallmark of aging. Indeed, older people present increasing rates and severity of bacterial and viral infections, cancer and reduced vaccine responses [2]. They suffer from more frequent and more severe community-acquired and nosocomial infections than younger people. They are at an increasing risk of adverse outcomes after hospitalization, such as a loss of function and independence, increased length of hospital stay, institutionalization and even death [2], [3].

The innate immune system is the first line of defense that protects host from invading pathogens. Pattern recognition receptors (PRR) such as toll-like receptors (TLR) recognize diverse pathogen-associated molecular patterns (PAMP) leading to the induction of inflammatory cytokines, chemokines and upregulation of co-stimulatory molecules [4]. TLR-dependent activation of antigen-presenting cells is a crucial step not only for the innate response but also for ensuing initiation of adaptive immune responses, in part through the induction of cytokine production. Interleukin (IL)-12p70 and IL-23 play crucial and distinct roles in shaping the innate and adaptive immune responses against invading pathogens. They are heterodimeric cytokines, composed of a common IL-12/23p40 subunit and a specific α-subunit (IL-12p35 and IL-23p19, respectively). IL-12p70 activates NK cells and favors the induction of Th1 CD4^+^ T cells and the effector function of CD8^+^ cytotoxic T cells, playing an important role in the protection against intracellular pathogens. IL-23 allows IL-17 and IL-22 production by innate lymphoid cells and the rapid recruitment of neutrophils. This cytokine is critical for the maintenance of Th17-type responses, intervening in the fight against extracellular pathogens [5]. TLR responses in old people have not been widely studied. The Leyden 85-plus study indicated that low IL-6, TNFα, IL-1β and IL-10 production upon LPS stimulation in whole blood was correlated with a 2-fold increase in overall mortality risk [6]. Furthermore, TLR-elicited cytokine production by circulating blood dendritic cells (DCs) was decreased in old individuals in comparison to healthy young controls [7], [8]. However, the influence of comorbidities and frailty in TLR responses is still unknown. Frailty is defined as a biological syndrome of decreased reserve and resistance to stressors resulting from cumulative decline across multiple physiologic systems (like osteopenia, sarcopenia, dyregulation of hypothalamic axis, heart rate variability …) thereby causing vulnerability to adverse outcomes. It is a distinct entity with multiple manifestations and no single manifestation by itself but clearly overlaps with age-associated comorbidities and disability [9]–[11]. Because of these complex interactions, when looking at immune functions in old age, it is necessary to integrate clinical parameters in order to identify correlative factors.

The aim of our study was to assess the relative impact of age, comorbidities and frailty on the capacity of blood cells to produce IL-12p70 and IL-23 in response to TLR ligation. We also assessed the potential interaction with biological parameters such as leucocyte telomere length (a marker of cellular senescence) or CMV status.

## Materials and Methods

### Subjects

Between 2010 and 2012, 108 subjects aged between 23 to 93 years (65 women and 43 men) were enrolled in this cross-sectional study. They were characterized for detailed medical history, ongoing comorbidities and routine blood tests (table 1). Comprehensive geriatric assessment was also screened in older individuals (>75 years, n = 52, table 2). The exclusion criteria were: C-reactive protein (CRP) ≥1 mg/dl, hepatic disturbance, presence of active cancer, autoimmune disease or infection, immunosuppression state, use of corticoids, immunosuppressors or non-steroid anti-inflammatory drugs (NSAID); advanced dementia (Mini Mental State Examination (MMSE) below 23 [12]) was also excluded for ethical reasons. Healthy young and old volunteers were recruited at the geriatric day center of tertiary Erasme hospital (Brussels) among hospital and laboratory employees, volunteers of a non-profit seniors association (Association pour le Soutien de l’Etude du Vieillissement) or through public solicitation. Hospitalized volunteers were recruited from cardiology, neurology, revalidation or endocrinology units or from the geriatric unit (tertiary Erasme hospital, Brussels).

**Table 1:** Characteristics of the study group.

	Entire group
N	100
Age (year)^1^	66.4 (23–93)
Gender M/F	35/65
BMI (kg/m^3^)^1^	24.9 (17–46)
Active smokers (%)	11
Hypertension (%)	50
Type 2 diabetes (%)	12
Hypercholesterolemia (%)	56
Cardiovascular diseases (%)	29
CMV seropositivity (%)	55

1 Median (range).

**Table 2:** Demographic, geriatric and biochemical characteristics of frail and non frail subjects >75 years of age.

	Non frail	Frail
N	27	25
Recruitment type	Ambulatory	Hospitalized
Age (years)^1^	80 (76–88)	83 (73–90)
Gender M/F	9/18	8/17
BMI (kg/m^2^)^1^	25.4 (19–32)	24.7 (18–34)
Active smokers (%)	0	0.08
Hypertension (%)	48	28
Type 2 diabetes (%)	0	28
Hypercholesterolemia (%)	74	60
Cardiovascular diseases (%)	3.7	60
Osteoporosis (%)	14.8	40
ISAR (score)	0 (0–1)	3 (2–4)
GDS (score)	1 (0–2)	5 (2–5)
Katz (score)	6 (6–7)	9 (7–12)
MMSE (score)	29 (28–29)	26 (24–28)
MNA (score)	26.8 (25.6–27.8)	20 (17.5–22.5)
CIRS-G (category number)	6 (3.5–6.5)	9 (7–10)
CIRS-G (global score)	9 (6.5–11)	19 (15–22)
CIRS-G (severity index)	1.6 (1.4–2)	2.2 (1,7–2,3)
Cholesterol (mg/dl)^1^	202 (182–223)	187 (173–206)
Ferritin (mg/dl) 1	107 (60–184)	176 (112–275)
Prealbumin (mg/dl)^1^	25 (22–28)	19 (17–24)
CMV seropositivity (%)	48	64

1 Median (range).

Flow cytometry experiments were conducted on additional subjects, either non frail (n = 10) or frail (n = 11) (table s1). Non frail old people were recruited at the geriatric day center of Erasme hospital among hospital and laboratory employees, volunteers of a non-profit seniors association (Association pour le Soutien de l’Etude du Vieillissement) or through public solicitation. One individual was excluded because he was considered as “prefrail” (MNA<23, see below). Frail patients were recruited in the geriatric unit (Erasme hospital) or in a nursing home (Sénieurie du Val, Wanze, Belgium). One subject was excluded because of CRP>1 mg/dl.

All patients were screened for significant underlying illness by direct questioning, medical archives and blood sampling (explained further).

### Ethics Statement

All subjects signed an informed consent and the study received approval from Erasme hospital Ethics Committee (808 route de Lennik, B-1070 Brussels, Belgium, agreation n°OM021).

### Clinical Characteristics

Social evaluation included determination of age, gender, home (private versus institution), and marital status. Clinical data comprised smoke and alcohol habits, pneumococcal and influenza vaccine status, allergy, body mass index, medical history, current treatment and reasons for hospitalization. Cardiovascular (CV) diseases were defined as history of stroke, myocardial infarct, cardiac insufficiency or failure, cerebral vascular disease, atheromatosis assessed by carotid or inferior member Doppler echography and ischemic symptoms. CV risk factors are defined as presence in the anamnesis of hypertension, type 2 diabetes, hypercholesterolemia, statin intake, infarct history and smoking.

For subjects >75 years, we performed a comprehensive geriatric assessment, based on several parameters. ISAR is a rapid scale performed at the emergency department which evaluates frailty with 6 questions about dependence, previous hospitalization, eye troubles, memory problems and number of medications [13]. The polypathology and the severity of the medical problems were scored using a cumulative illness rating scale (CIRS-G). It is an instrument to quantify disease burden because of its ability to differentiate older adults with the highest risk and severity of infection with a markedly impaired vaccine response [14]–[16]. It comprises a comprehensive review of medical problems of 14 organ systems. It is based on a 0 to 4 rating of each organ system [17], [18]. The geriatric depression scale was used to assess the probability of a depressed mood (GDS-15) in 15 questions [19], [20]. The assessment of “activities of daily living” (ADL) was made by using Katz’s scale. It includes the following items: bathing, dressing, transfer, toilet, continence and eating. Each task is graded on a 3-level scale (1 to 3 for Katz’s scale), where lower levels represent the absence of dependence and upper level the maximal dependence for the task [21]. Cognitive functions were assessed using the Mini Mental State Examination (MMSE). Possible scores range from 0 to 30 points, with lower scores indicating impaired cognitive function [12]. Nutritional status was assessed using the mini nutritional assessment (MNA). A score ≥24 points identifies patients with a good nutritional status. Scores between 17 and 23.5 points identify patients at risk for malnutrition [22], [23]. These patients have not yet started to lose weight and do not show low plasma albumin levels but have lower protein-caloric intakes than recommended. A score <17 points indicates protein-caloric malnutrition. Pain was assessed using a visual analogical scale from 0 to 10 points.

We performed routine biochemical assessment to identify potential exclusion factors. CMV-specific IgG levels were determined by ELISA (ETI-CYTOK-GPLUS; Diasorin, P002033).

### Sample Collection and Management

Venous blood samples (55 ml) from all subjects were collected in pyrogen-free, heparinized tubes between 11.00 and 13.00. For *ex vivo* stimulation with TLR agonists, blood was diluted 2-fold with sterile RPMI-1640 (Lonza BE12-115F) and incubated in the presence of *E. coli*-derived lipopolysaccharide 20 ng/ml (LPS, invivogen), Resiquimod 10 µg/ml (R848, Pharmatech labs) or LPS+R848 at 37°C and 5% CO2. After 18 h, supernatants were collected and stored at −80°C to measure cytokine production by ELISA (duoset or quantikine kits, R&D systems). In an additional group of older subjects, we assessed surface expression of costimulatory molecules on monocytes and DC subsets. For this purpose, 400 µl of whole blood were incubated with PBS or TLR ligands for 5 hours. For intracytoplasmic cytokine stainings, brefeldin A (15 µg/ml, sigma, B7651-5MG) was added during the culture. Red blood cells were lyzed by incubation in FACS cell lysing buffer (BD Biosciences). Cells were then frozen at −80°C.

For phosphoprotein stainings, 400 µl of whole blood were stimulated or not with TLR ligands during 20 minutes. Cells were fixed with 10% Formaldehyde UP (EM grade polysciences, 04018) and then incubated with triton X100 (0.233%, Sigma, T-9284). Cells were then washed and frozen at −80°C in 400 µl freezing buffer (20% FCS, 10% glycerol and 70% RPMI).

For telomeres quantification, peripheral blood mononuclear cells (PBMCs) were also separated using standard density gradient centrifugation over Ficoll-Plaque (lympoprep TM, Axis shield). DNA from these PBMC was isolated using Wizard SV genomic DNA purification system (Promega) according to the manufacturer’s recommendations.

### Telomeres Analyses

The measure of relative telomere length was performed by real time quantitative PCR as previously described [24].

### Flow Cytometry

For the relative quantification of DC and monocyte subsets and the expression of costimulatory molecules, 400 µl of blood was fixed and lyzed for 10 min using FACS cell lysing buffer (BD biosciences, ref 349202). Cells were frozen at −80°C in this buffer until completion of the recruitment. After thawing, cells were incubated with human Fcblock (Miltenyi, 130-059-901). Cells were stained with the following antibodies: Mix 1 (for DCs) : anti-CD3 FITC (BD, 345764), CD19 FITC (BD, 345788), CD56 FITC (BD, 345811), HLA-DR PerCP (BD, 347402), CD14 APC-H7 (BD, 641394), CD11c APC (BD, 333144), CD86 PB (BD, 560357), CD54 PE (Biolegend, 322708), TLR4 AF700 (eBioscience, 56-9917-42) and CD123 PeCy7 (eBioscience, 25-1239-42). Mix 2 (for monocytes): anti-CD80 FITC (BD, 557226), HLA-DR PerCP, CD14 PB (BD, 558121), CD11c APC, CD16 PE (BD, 559331), TLR4 AF700 (eBioscience, 56-9917-42). Flow cytometric analyses were performed on a LSRII flow cytometer (BD biosciences). Conventional myeloid DCs were defined as: Lin (CD19/CD3/CD56/CD14)^−^, HLA-DR^+^, CD11c^+^CD123^−^. Plasmacytoid DCs were defined as: Lin (CD19/CD3/CD56/CD14)^−^, HLA-DR^+^, CD11c^−^CD123^+^ cells. Monocyte subsets were all HLA-DR^+^CD11c^+^ and differentiated according to expression of CD14 and CD16: “classical” monocytes (CD14^++^CD16^−^) “intermediate” monocytes (CD14^+^CD16^+^) and “non-classical” monocytes (CD14^dim^, CD16^++^). Samples without antibodies against CD54 and CD86 were used as controls.

For intracellular cytokine stainings, cells were permeabilized with 500 µl of permeabilization buffer 2 (BD, 340973). Cells were then stained with the following antibodies (Mix3): CD3 FITC, CD19 FITC, CD56 FITC, HLA-DR PerCP, CD14 APC-H7, CD11c APC, IL-6 PE (BD, 559331) IL-12p40 PB (eBioscience, 48-7129-41) and TNFα AF700 (BD, 557996). Samples without antibodies against cytokines (IL-6, TNFα and IL-12/23p40) were used as controls.

For phosphoprotein stainings, cells were permeabilized with 50% methanol. Cells were then stained with the following antibodies (Mix4-5): CD3 FITC, CD19 FITC, CD56 FITC, HLA-DR PerCP, CD14 PB, CD11c AF700 (BD, 5-61352), phospho-p65 AF648 (BD, 558422) or phospho-p38 AF648 (BD, 612595) and phospho-Erk PE (BD, 612566). For each subset and stimulation, MFI (mean of fluorescence intensity) for p65, p38 and Erk phosphorylation were assessed. Samples without antibodies against phosphoprotein (p65, p38, Erk) were used as control.

### Statistics

The SigmaStat®software package version 3.5 (Jandle Scientific) was used for multivariate analyses and GraphPad prism 5® software for univariate analyses and Mann-Whitney rank sum test. Univariate analyses were depicted by Pearson’s coefficient. Cytokine levels were normalized to monocytes counts. Independent factors for cytokines levels in univariate analyses for all volunteers comprised age, gender, CV diseases and risk factors, smoke, statin, antidepressant intake, CMV status, telomere length. Independent factors for cytokines levels in univariate analyses for subjects >75 years comprised the above mentioned factors, all geriatric scales and osteoporosis.

Multi-linear regression analyses were tested using a stepwise backward selection of the explicative variables. Katz, ISAR, MNA and clinical variables were treated as dichotomous variables while other data were continuous with frailty cut off of ≥8, <23.5, >1 respectively. We used Mann-Whitney rank sum test to compare geriatric traits, TLR responses in non-frail and frail older individuals (ELISA and cytometry data). A probability level of p<0.05 was considered to be significant.

## Results

### Characteristics of the Enrolled Individuals

100 subjects were included in the analysis and 8 subjects were excluded because CRP values exceeded 1 mg/dl. The study was designed to include both healthy and chronically affected subjects ranging from 23 to 93 years of age in order to distinguish impact of age, comorbidities and frailty. Fifty two subjects were older than 75 years (27 non-frail subjects, 25 frail subjects assessed by ISAR>1 point). The demographic, comorbidities, treatment and biochemical characteristics of the entire group are presented in table 1. As geriatric scales are only validated in individuals above 75 years of age, we analyzed the influence of frailty on TLR responses in age group. The demographic, clinical and biochemical characteristics of non-frail and frail older subjects are presented in table 2. As expected, in patients with ISAR>1 point, decreased grip strength, depression states (GDS), comorbidity burden (CIRS-G), dependence (Katz scale), cognitive troubles (MMSE) and impaired nutritional status (MNA) were more prevalent in comparison to non-frail individuals.

### Predictive Factors for TLR Responses in the Whole Study Group

In order to study the capacity of whole blood cells to produce bioactive IL-12p70 and IL-23, we first assessed the optimal experimental conditions. As previously described in other systems [25], we observed that a combination of TLR4 (LPS) and TLR7/8 (R848) ligands induced robust production of both cytokines. This effect was synergistic for IL-12p70 and additive for IL-23 (data not shown). We therefore selected this experimental condition to define the influence of clinical factors on IL-12p70 and IL-23 production in our study group. We performed univariate analyses to assess potential association between cytokine levels on one hand and age, CV risk factors and diseases, gender, CMV status or telomere length on the other hand. No significant association was found between IL-12p70 or IL-23 levels for age, gender, CV risk factors and diseases, CMV status and telomere length.

### Predictive Factors for TLR Responses in Aged Individuals

**Figure 1:**
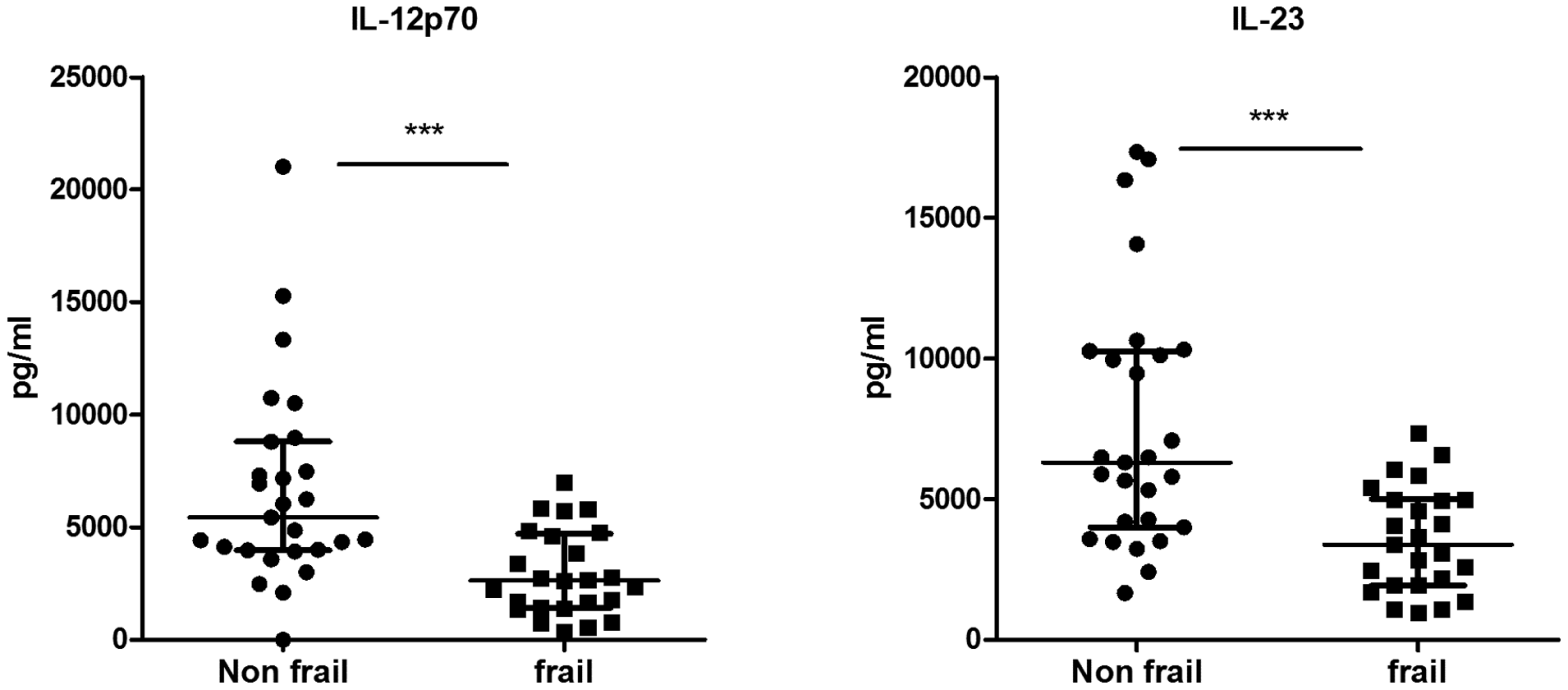
Decreased production of IL-12p70 and IL-23 in response to LPS+R848 stimulation in frail old individuals. Whole blood cells from frail (n = 10) and non frail (n = 9) old individuals were stimulated with LPS+R848 during 18 h. IL-12p70 and IL-23 were measured in cell-free supernatants by ELISA. Each dot represents a single donor and the bars represent median values ± interquartile range. ***p<0.001.

Next, we assessed the potential association between cytokine production upon TLR stimulation and geriatric clinical scales. As these scales are only validated in aged individuals, we restricted our analysis to subjects above 75 years of age. First we compared IL-12p70 and IL-23 response upon LPS+R848 stimulation between frail and non-frail old individuals (figure 1). We observed that production of both cytokines was significantly decreased in frail patients. Secondly, we performed univariate analyses to assess the association between geriatric scales and these cytokines responses (Table 3). Frailty (ISAR), dependence (Katz), poor nutritional status (MNA) and comorbidities (CIRS-G, global score) were significant predictive factors for IL-12p70 and IL-23 production in response to LPS+R848 combination. Depression, assessed by GDS and prealbumin levels were only significantly correlated with TLR-stimulated IL-23 (there was a trend for prealbumin levels and IL-12p70 production). We performed multivariate analyses for both cytokines to adjust for age, gender and CV risk factors and diseases. Because geriatric scales influence each other, each geriatric scale (ISAR, Katz, MNA, GDS) was included separately (table 4). Geriatric scales remained significantly associated with cytokine levels in these analyses. This was not the case for prealbumin levels anymore. From these results, we concluded that production of IL-12p70 and IL-23 in response to LPS+R848 was not affected by age or CV comorbidities. Furthermore, we didn’t find any clear association with other biological markers such as telomere length of PBMCs or CMV status. In contrast, frailty, dependency and poor nutritional status were strongly associated with impaired IL-12p70 and IL-23 production.

**Table 3:** Association between TLR response and frailty (N = 52): Univariate analyses.

Cytokine	Stimulation	Gender	CV risk factors	CV diseases	ISAR	GDS	Katz	MNA	CIRS-G	Prealbumin
IL-12	LPS+R848	NS	NS	NS	p = 0.0006R^2^ = 0.21	NS	p = 0.0065R^2^ = 0.13	p = 0.0045R^2^ = 0.15	p = 0.006R^2^ = 0.14	p = 0.08
IL-23	LPS+R848	NS	NS	NS	p = 0.0003R^2^ = 0.23	p = 0.048R^2^ = 0.08	p = 0.005R^2^ = 0.14	p = 0.0008R^2^ = 0.2	p = 0.003R^2^ = 0.22	p = 0.0065 R^2^ = 0.14

**Table 4:** Association between TLR response and frailty (N = 52) : Multivariate analyses.

N = 52	LPS+R848-induced IL-23	LPS+R848-induced IL-12p70
Model 1	ISAR R^2^ = 0.23; F = 15; p<0.001; standardized coefficient = −0.48	ISAR R^2^ = 0.21; F = 13.5; p<0.001; standardized coefficient = −0.46
Model 2	Katz R^2^ = 0.14; F = 8.4; p = 0.005; standardized coefficient = −0.38	Katz R^2^ = 0.14; F = 8; p = 0.007; standardized coefficient = −0.37
Model 3	MNA R^2^ = 0.21; F = 12.7; p<0.001; standardized coefficient = −0.45	MNA R^2^ = 0.15; F = 8.87; p = 0.004; standardized coefficient = −0.39
Model 4	NS	NS
Model 5	R^2^ = 0.16; F = 4.6 GDS p = 0.009; standardized coefficient = −0.38gender p0.032; standardized coefficient = −0.3;	–

Model 1: Adjusted for ISAR score, age, gender, CV risk factors and diseases.

Model 2: Adjusted for Katz score, age, gender, CV risk factors and diseases.

Model 3: Adjusted for MNA score, age, gender, CV risk factors and diseases.

Model 4: Adjusted for prealbumin, age, gender, CV risk factors and diseases.

Model 5: Adjusted for GDS, age, gender, CV risk factors and diseases.

### Decreased IL-12/23p40 Expression in Conventional Dendritic Cells from Frail Individuals

**Figure 2:**
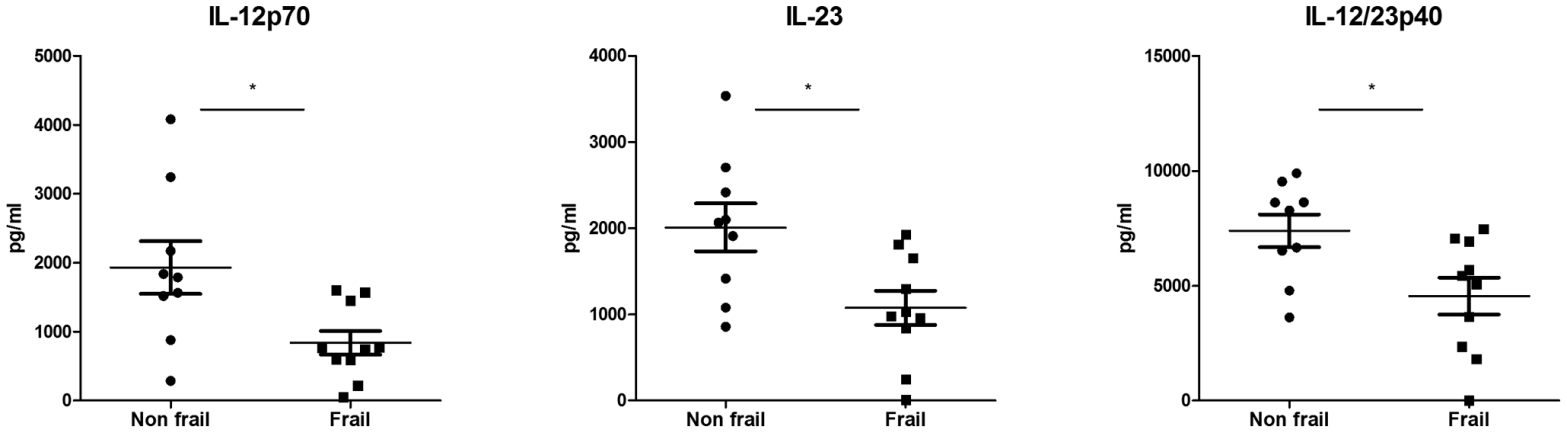
Decreased production of IL-23, IL-12p70 and IL-12/23p40 in response to LPS+R848 stimulation in a subgroup of frail old individuals. Whole blood cells from frail (n = 10) and non frail (n = 9) old individuals were stimulated with LPS+R848 during 24 h. IL-12/23p40, IL-12p70 and IL-23 were measured in cell-free supernatants by ELISA. Each dot represents a single donor and the bars represent median values ± interquartile range. *p<0.05.

In order to gain insight into the mechanisms that could account for these observations, we performed additional experiments in a subgroup of aged subjects (>75 years). For this purpose, we recruited 9 non-frail old subjects and 10 frail old patients (identified by ISAR score>1 point, MNA score <23 points and KATZ score ≥8 points). Demographic, comorbidities, biological data and geriatric scales are shown in table S1. Once again, depression state (GDS), comorbidity burden (CIRS-G), and cognitive troubles (MMSE) were also more severe in these frail patients in comparison to non-frail individuals. However, prealbumin and cholesterol levels were not decreased in the frail group, suggesting that these frail patients were potentially at risk for malnutrition but not malnourished. We confirmed that IL-23 and IL-12p70 production in response to LPS+R848 combination was significantly decreased in this subgroup of frail individuals in comparison to non frail subjects (figure 2). Along this line, production of IL-12/23p40, the common subunit of IL-12p70 and IL-23, was also decreased in this subgroup.

**Figure 3:**
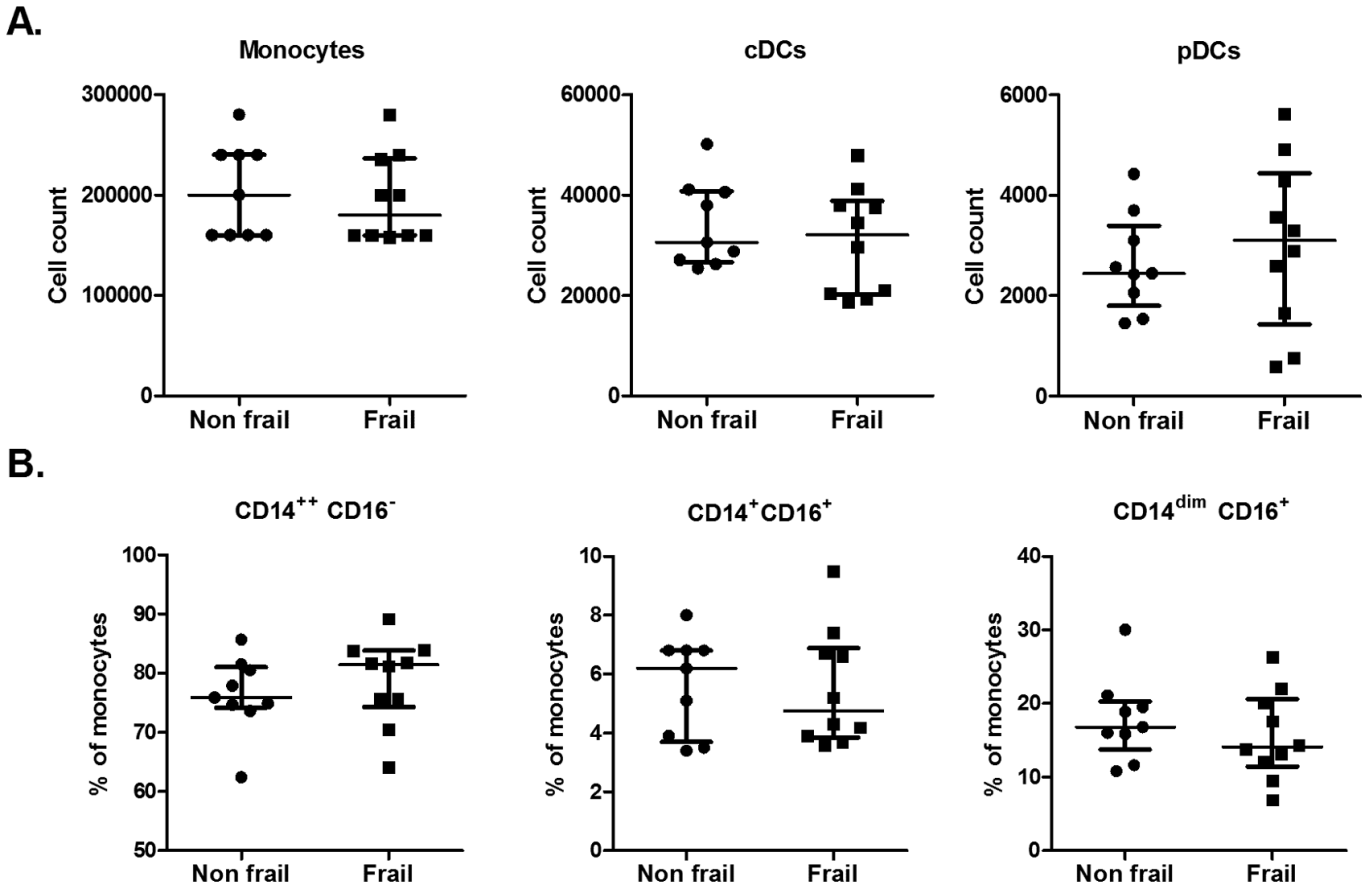
Monocytes and DC subsets in a subgroup of non frail and frail old subjects. Whole blood cells were immediately fixed and stored in FACS cells lysing buffer after blood collection. A) Absolute cell counts of monocytes (Lineage^−^HLA-DR^+^CD11c^+^CD14^+^), cDCs (Lineage^−^HLA-DR^+^CD11c^+^CD14^−^CD123^−^) and pDCs (Lineage^−^HLA-DR^+^CD11c^−^CD14^−^CD123^+^). B) Relative proportion of monocyte subsets: CD14^++^CD16^−^, CD14^+^CD16^+^ and CD14^dim^CD16^+^. Each dot represents a single donor and the bars represent median values ± interquartile range.

**Figure 4:**
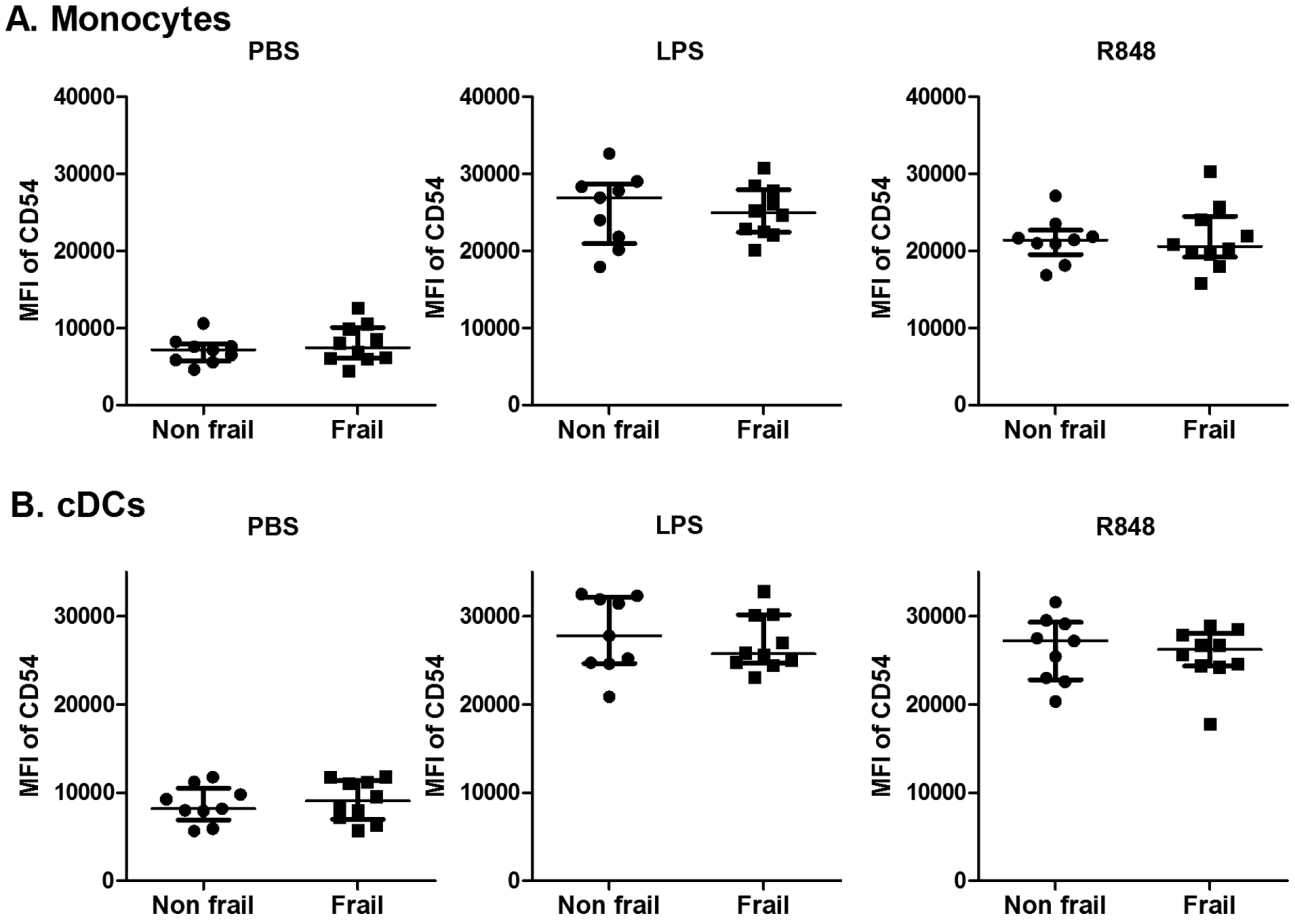
Activation state (CD54) of cDCs and monocytes in non frail and frail old subjects. Whole blood cells were stimulated or not with LPS or R848 during 5 h. Expression of CD54 was assessed for (A) monocytes (Lineage^–^HLA-DR^+^CD11c^+^CD14^+^), (B) cDCs (Lineage^−^HLA-DR^+^CD11c^+^CD14^−^CD123^−^). MFI (mean) values for each donor are shown. The bars represent median values ± interquartile range.

**Figure 5:**
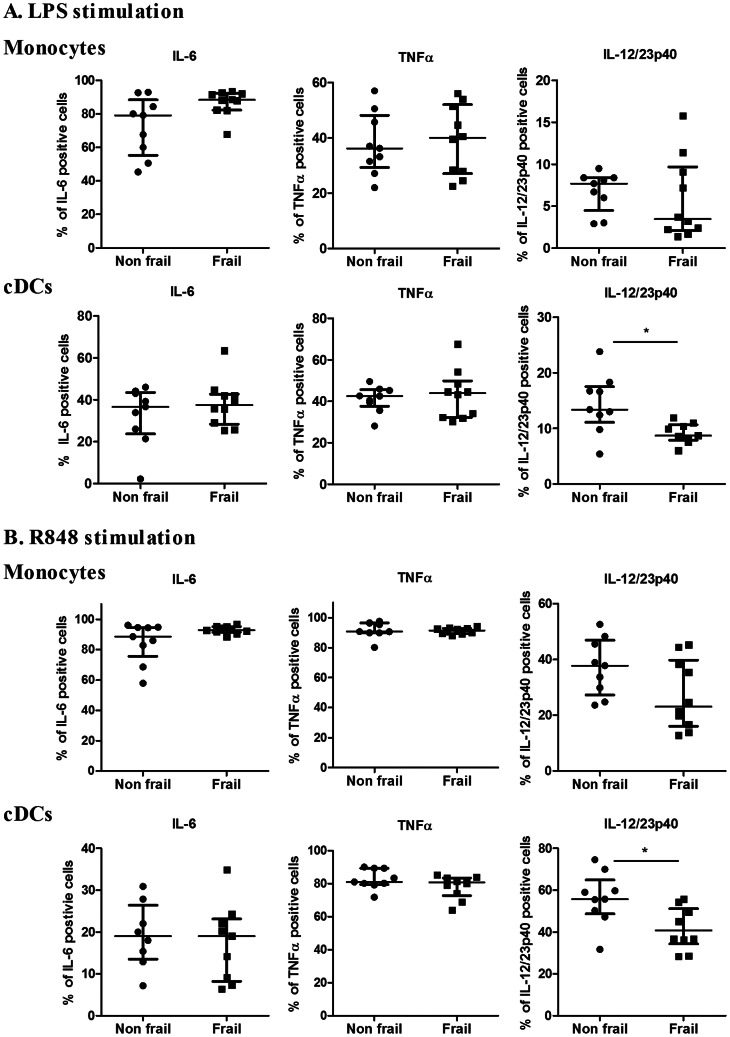
Intracellular cytokine staining of cDCs and monocytes from non frail and frail old subjects. Whole blood cells were stimulated with (A) LPS or (B) R848 during 5 h in presence of brefeldin A. Percentage of IL-6^+^, TNFα^+^ and IL-12/23p40^+^ monocytes and cDCs are shown. The bars represent median values ± interquartile range. *p<0.05.

**Figure 6:**
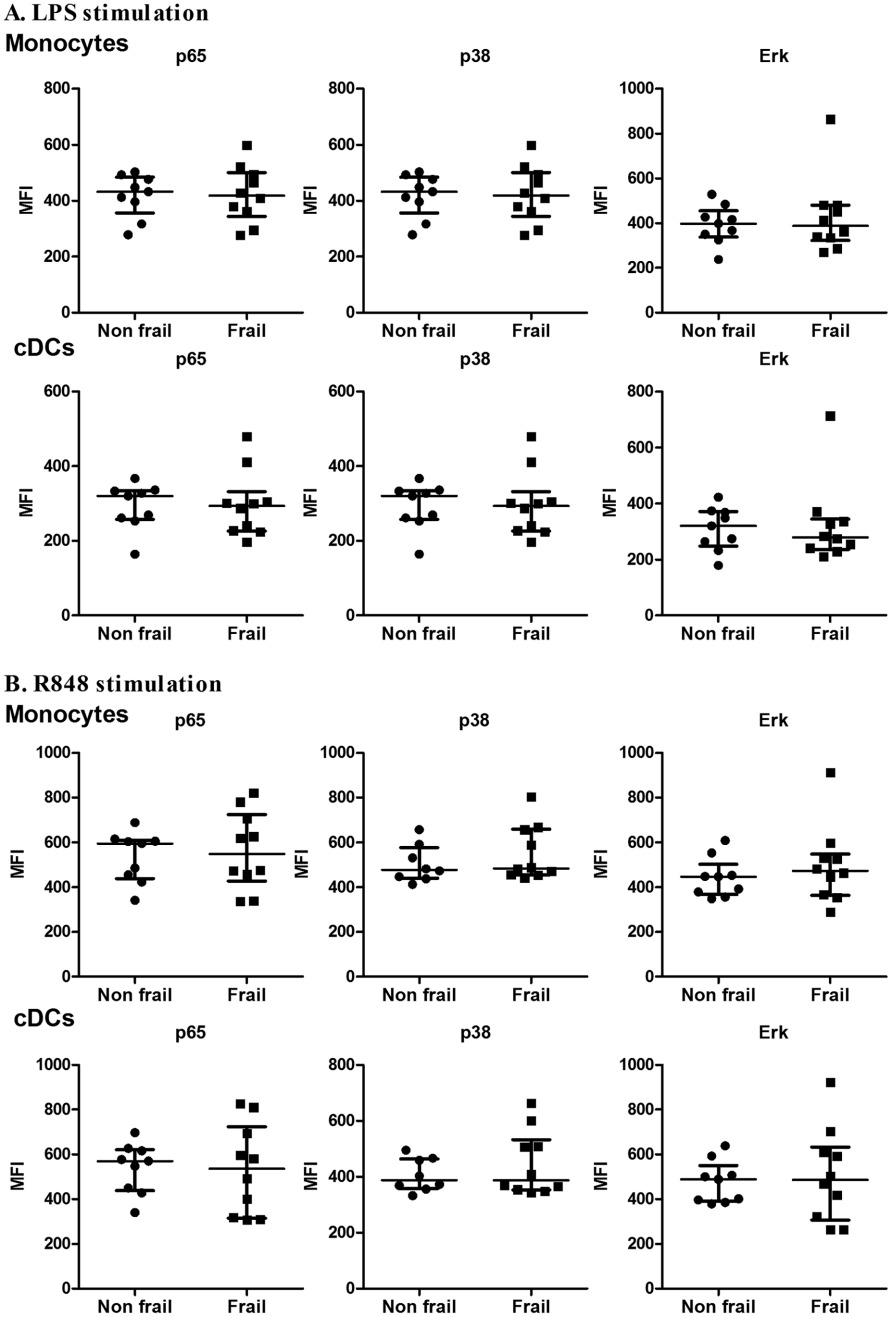
NF-κB p65, p38 and Erk phosphorylation of monocytes and cDCs after LPS and R848 stimulation in non frail and frail old subjects. Whole blood cells were stimulated with (A) LPS or (B) R848 for 20 minutes. MFI (mean) expression of phosphorylated NF-**κ**B p65, p38 and Erk MAPK protein in monocytes (Lineage^–^HLA-DR^+^CD11c^+^CD14^+^) or cDCs (Lineage^−^HLA-DR^+^CD11c^+^CD14^−^) are shown for each donor. Bars represent median values ± interquartile range. *p<0.05.

We first assessed the absolute and relative cell counts of the main subsets of monocytes (CD14^++^/CD16^−^, CD14^+^/CD16^+^, CD14^dim^/CD16^+^) and dendritic cells (conventional and plasmacytoid) (cDCs and pDCs) in these 2 groups (see figure 3). Cellular composition in frail and non frail individuals was found to be comparable, indicating that impaired IL-12 and IL-23 induction in frail patients was not due to decreased numbers of monocytes or DC subsets. Furthermore, expression of TLR4 at the surface of monocytes and cDCs and was found to be comparable (data not shown). In order to further define the events downstream of TLR4 and TLR7/8, we performed stimulation with single ligand. We assessed the expression of co-stimulatory molecules (CD80, CD86 and CD54) and HLA-DR on CD14^+^ monocytes and conventional DCs in response to either LPS or R848. Basal expression and upregulation of these markers was found to take place to a similar extend in both groups (Fig. 4, fig. S1 and S2). Along this line, we observed that the capacity of these cellular subsets to produce IL-6 and TNFα in response to LPS or R848 stimulation (assessed by intracytoplasmic stainings) was comparable in both groups (figure 5). In contrast, cDCs from frail patients present a significant decrease in IL-12/23p40 production upon either LPS or R848 stimulation. For the same donors, production of IL-12/23p40 by CD14^+^ monocytes was found to be comparable in both groups. These results indicate that impaired IL-12 and IL-23 production in frail old patients is reflected by a selective perturbation of the induction of the common IL-12/23p40 chain by cDCs. Finally, we looked at early TLR-induced events in these cellular subsets. In whole blood assays, we observed that LPS and R848 rapidly induced the phosphorylation of Erk and p38 MAP kinases and of NF-κB p65 transcription factor (assessed by phosflow techniques) in healthy volunteers (figure S3). We compared these parameters at the peak of the response (20 min post-stimulation) in frail and non frail individuals. As shown in figure 6, these proximal TLR-induced signaling events were found to be largely comparable in both groups.

Taken together, our results indicate that impaired TLR-stimulated IL-12 and IL-23 production is associated with frailty and results from altered blood cDCs function. Importantly, proximal TLR signaling pathways and other downstream events (upregulation of costimulatory markers, TNFα and IL-6 expression) were not found to be compromised in these cells.

## Discussion

In this study, we show that frailty and not age by itself affected IL-12p70 and IL-23 production upon TLR stimulation. Conventional DCs from frail geriatric patients displayed an impaired capacity to produce IL-12/23p40. However, proportion of DCs and monocytes subsets, phenotypic maturation and proximal signaling events were found to be comparable in frail and healthy old subjects. Several reports have examined LPS-induced cytokine production in PBMCs or whole blood from old subjects yielding conflicting results [26]–[28]. As most of these studies did not take into account geriatric clinical characteristics, it is difficult to address the specific role of age in these observations. We observed that in old patients, frailty and associated dependence and malnutrition were strongly correlated with reduced IL-12p70 and IL-23 production upon LPS and R848 stimulation. Interestingly, in patients with poor nutritional status, this was not limited to these cytokines as we also observed decreased IL-6 and TNFα production upon LPS or R848 stimulation (data not shown). In line with this observation, in the Leyden 85+ study, reduced responsiveness to LPS stimulation (lower TNFα, IL-1β, IL-6, IL-10 and IL-1Ra production by whole blood cells) from community-dwelling or nursing home 85-year old residents was significantly associated with a worse survival and risk factors like history of malignancies, chronic illness and elevated CRP levels [6]. Moreover, Corsini *et al.* showed that functional old people displayed a decreased TNFα and increased IL-10 production in response to LPS compared to young people. These immunological characteristics likely contribute to the predicted low responses to influenza vaccination in old people [29]. Interestingly, in the present study, IL-10 production upon TLR stimulation was comparable in frail and non frail geriatric patients (data not shown) arguing in favor of the hypothesis that increased or unchanged anti-inflammatory status and decreased proinflammatory status play a role in infection susceptibility. Taken together, we suggest that alterations of TLR-induced inflammatory cytokines production in old age could be a hallmark of frailty. We found that production of both IL-12p70 and IL-23 were the most severely affected in frail patients. Some reports indicate that there is a general decrease of peripheral blood pDC and cDC number with age but this remains controversial [7], [8], [30]–[35]. In a small group of aged subjects, we did not find any differences between non frail and frail patients in the absolute or relative numbers of monocytes or DCs subsets. Human monocytes from aged individuals present lower surface TLR1 and TLR4 expression than their younger counterparts [36], [37]. We did not observe any differences in TLR4 expression or TLR-induced upregulation of HLA-DR and costimulatory molecules on monocytes and cDCs. Furthermore, TLR4 expression and proximal TLR signaling events, leading to Erk and p38 MAP Kinase and NF-**κ**B activation were also comparable in healthy and frail groups, indicating that other molecular events, such as activation of transcription factors from the interferon regulatory factors (IRFs) could be implicated in these observations. We showed that impaired IL-12p70 and IL-23 production in frail patients was associated with a poor capacity of cDCs rather than monocytes to produce IL-12/23p40. Recent reports indicate that cDCs from old individuals displayed reduced expression of IL-12/23p40 in comparison to younger controls [7], [8]. This decrease in TLR-induced cytokine production was strongly associated with the inability to mount protective antibody responses to influenza vaccine. Our results suggest that this is not a direct consequence of aging but that additional age-associated clinical parameters could be implicated.

Poor nutritional status in older patients increases the risk of frailty [10], [11]. Indeed, higher protein consumption reduces the incidence of frailty in older women and in very old people [38] while low MNA score is correlated with poor outcome at hospitalization [39]. Worse ADL and nutritional status are also correlated with poor influenza vaccination in older patients [40] and whey protein supplements have been shown to boost immunological response to pneumovax in older people [41]. Older individuals that benefit from micronutrients supplementation (zinc, antioxidant, selenium, oligosaccharides…) showed a decrease in the severity of upper respiratory tract infections and an increased responsiveness to vaccination [42], [43]. Malnourished mice challenged with LPS *in vivo* also presented a decrease in serum TNFα and IL-1β levels, indicating that nutritional status can impact innate immune responses [44]. Hence, we suggest that poor nutritional status could be an important contributing factor to the decreased TLR responsiveness in frail patients.

Our findings have important clinical implications as IL-12p70 and IL-23 play an important role in the defense against intracellular and extracellular pathogens, respectively. Hence, decreased capacity to produce these cytokines in frail geriatric patients could account for their susceptibility to pathogens such as M. *tuberculosis*, C. *difficile* or S. *pneumoniae* infections. Further studies will be necessary to understand the underlying causes responsible for impaired DC function in frail geriatric patients.

## Supporting Information

Figure S1 **Activation state (CD86 and HLA-DR) of monocytes and cDCs in non frail and frail old individuals.** Whole blood cells were stimulated or not with LPS or R848 during 5 h. Expression of CD86 (A) and HLA-DR (B) was assessed for monocytes (Lineage^−^HLA-DR^+^CD11c^+^CD14^+^), cDCs (Lineage^−^HLA-DR^+^CD11c^+^CD14^−^CD123^−^). MFI (mean) values for each donor are shown. The bars represent median values ± interquartile range.
(TIF)

Figure S2 **CD80 basal expression on different monocytes subsets in non frail and frail old individuals.** Whole blood cells were directly fixed after blood collection. Expression of CD80 was assessed for classical monocytes (Lineage^–^HLA-DR^+^CD11c^+^CD14^++^CD16^−^), intermediate monocytes (Lineage^–^HLA-DR^+^CD11c^+^CD14^+^CD16^+^) and inflammatory monocytes (Lineage^–^HLA-DR^+^CD11c^+^CD14^dim^CD16^+^). MFI (mean) values for each donor are shown. The bars represent median values ± interquartile range.
(TIF)

Figure S3 **Induction of NF-κB p65, p38 and Erk phosphorylation in monocytes and cDCs after LPS or R848 stimulation.** Whole blood cells from healthy old volunteers were incubated with PBS alone or the indicated TLR ligand for 20 minutes. MFI (mean) values for phosphorylated protein in monocytes (Lineage^–^HLA-DR^+^CD11c^+^CD14^+^) or cDCs (Lineage^−^HLA-DR^+^CD11c^+^CD14^−^) are shown. One representative donor of 10 is shown.
(TIF)

Table S1 **Characteristics of subgroup of individuals >75 years.**
(DOCX)
