# Supplementary material for: Frailty in Old Age Is Associated with Decreased Interleukin-12/23 Production in Response to Toll-Like Receptor Ligation
Source: PLoS One. 2013 Jun 5;8(6):e65325. doi: 10.1371/journal.pone.0065325 (PMC3673922; doi:10.1371/journal.pone.0065325)
Supplement: Table S1 — Characteristics of subgroup of individuals >75 years. (DOCX) [file pone.0065325.s004.docx]

**Supplementary material**

**Table S1 : Characteristics of subgroup of individuals > 75 years**

**a) Demographic characteristics and comorbidities**

|  | Non frail | Frail |
| --- | --- | --- |
| N | 9 | 10 |
| Recruitment type | Ambulatory | Ho Hospitalized or Nursing home |
| Age^1^ | 80 (75 - 85) | 86.4 (80 - 92) |
| Gender M/F | 4/5 | 2/8 |
| BMI^1^ | 26 (20 - 30) | 21.5 (12.5 - 27) |
| Active smokers | 0 % | 0 % |
| Hypertension | 66.7 % | 99 % |
| Type 2 diabetes | 0 % | 10 % |
| Hypercholesterolemia | 100 % | 60 % |
| Cardiovascular diseases | 55 % | 50 % |
| Osteoporosis | 22 % | 30 % |

^1^Median (range)

**b) Geriatric characteristics**

|  | Non frail | Frail |
| --- | --- | --- |
| ISAR score | 0.5 (0  - 1) | 4 (2 - 4) *** |
| GDS score | 0 (0 - 1) | 4.5 (1 - 10) ** |
| Katz score | 6 (6 - 7) | 11 (8 - 17) ** |
| MMSE score | 29 (27 - 29) | 25.5 (12 - 28) ** |
| MNA score | 27.5 (25 - 29) | 17 (12.5 – 22.5) *** |
| CIRS-G (category number) | 5 (3 - 8) | 7.5 (6 - 9) ** |
| CIRS-G (global score) | 9 (2 - 15) | 17 (14 - 25) *** |
| CIRS-G (severity index) | 1.8 (1 – 2.3) | 2,3 (1.9 – 2.8) ** |

Median (range)

**p<0.01; ***p<0.001

**c) Biochemical characteristics**

|  | Non frail | Frailty |
| --- | --- | --- |
| Cholesterol (mg/dl) ^1^ | 188 (154 - 220) | 190 (105 - 270) |
| Ferritin (mg/dl) ^1^ | 165 (35 - 335) | 86 (25 - 417) |
| Prealbumin (mg/dl) ^1^ | 26 (24 - 31) | 23.5 (14 - 34) |
| CMV seropositivity (%) | 55 | 80 |

^1^Median (range)
